# Age‐Dependent Effects of Muscle Resting Calcium on Fasting Blood Glucose: Implications for Prediabetes Risk

**DOI:** 10.1002/edm2.70052

**Published:** 2025-05-06

**Authors:** Eshwar R. Tammineni, Lourdes Figueroa, Michael Fill, Sheila Riazi, Carlo Manno

**Affiliations:** ^1^ Department of Physiology and Biophysics Rush University Medical Center Chicago Illinois USA; ^2^ Malignant Hyperthermia Unit, Department of Anesthesiology and Pain Medicine University Health Network, University of Toronto Toronto Ontario Canada

**Keywords:** FBS levels, resting cytosolic calcium, skeletal muscle, type 2 diabetes

## Abstract

**Background and Aims:**

Skeletal muscle is the primary site for insulin‐mediated glucose uptake and is critical in maintaining whole‐body glucose homeostasis. Muscle cells from malignant hyperthermia‐susceptible (MHS) individuals exhibit elevated resting cytosolic calcium concentration ([Ca^2+^]_cyto_), and MHS subjects have a higher incidence of hyperglycaemia. This study investigates the association between elevated resting [Ca^2+^]_cyto_ and fasting blood sugar (FBS) levels while accounting for subject demographics and clinical variables.

**Methods:**

We measured resting [Ca^2+^]_cyto_ in myotubes derived from muscle biopsies of control and MHS subjects. We analysed the impact of [Ca^2+^]_cyto_ on FBS levels based on age, sex, and MH status through correlation and comparative analyses. Data were stratified by FBS, [Ca^2+^]_cyto_, and age, and heat map and 3D mesh plot analyses were performed to assess the risk of prediabetes in subjects with varying [Ca^2+^]_cyto_ and age.

**Results:**

Between 2013 and 2024, muscle biopsies from 152 subjects (90 MHS, 62 controls) were used to establish primary myotube culture. MHS myotubes exhibited significantly higher resting [Ca^2+^]_cyto_ than controls and [Ca^2+^]_cyto_ positively correlated with fasting blood sugar (FBS) in MHS subjects (*r* = 0.227; *p* = 0.031) and across the entire cohort (*r* = 0.176; *p* = 0.034). This correlation was high in prediabetic individuals (*r* = 0.43; *p* = 0.005) but absent in those with normal FBS. Subjects over 40 years with [Ca^2+^]_cyto_ > 150 nM showed a higher risk of prediabetes. While aging is a significant risk factor for type 2 diabetes, increased age did not impact FBS levels in individuals with [Ca^2+^]_cyto_ below 150 nM. However, in those with [Ca^2+^]_cyto_ exceeding 150 nM, increasing age significantly influenced FBS.

**Conclusions:**

Elevated resting [Ca^2+^]_cyto_ in skeletal muscle amplifies the risk of hyperglycemia and may contribute to the onset of type 2 diabetes.

## Graphical abstract

This correlation study shows that elevated resting calcium levels in skeletal muscle are associated with an increased risk of developing diabetes in an age‐dependent manner.

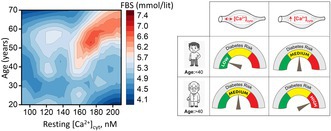

## 1 Introduction

Type 2 diabetes (T2D) is a major global health issue, affecting quality of life and economic stability worldwide. Its development is characterised by sequential progression from insulin resistance in key insulin‐sensitive tissues to impaired insulin secretion caused by pancreatic beta cell dysfunction [1]. Early intervention, targeting the initial defects in this process, is critical for preventing the full onset of T2D. Notably, insulin resistance in skeletal muscle is among the earliest detectable abnormalities [1]. A deeper understanding of the molecular mechanisms driving skeletal muscle insulin resistance (IR) is essential for developing therapeutic strategies to prevent or slow early disease progression. Recent studies on diabetic mouse models have highlighted that chronically elevated basal cytosolic calcium in skeletal muscle fibres is a significant factor disrupting glucose uptake, leading to hyperglycemia [2]. Treatment with the muscle relaxant dantrolene, which lowers [Ca^2+^]_cyto_, has been proposed as a promising intervention that improves glucose uptake and alleviate hyperglycemia [2].

Malignant hyperthermia (MH) is a rare pharmacogenetic disorder of skeletal muscle, triggered in susceptible individuals by volatile anaesthetics, succinylcholine, and, less commonly, by strenuous exercise or environmental heat [3]. Even in the absence of these triggers, most MH‐causing mutations in excitation‐contraction (EC) coupling proteins (‘couplon’) enhance sarcoplasmic reticulum (SR) calcium leak, leading to elevated [Ca^2+^]_cyto_ levels [4, 5, 6, 7]. This abnormal calcium handling in MHS subjects has been linked to an increased risk of hyperglycaemia and T2D [8, 9]. Recent studies showcase the advantages of MH mice as models for defining the impact of elevated [Ca^2+^]_cyto_ on skeletal muscle insulin resistance [2, 8]. Our previous research has demonstrated that sustained [Ca^2+^]_cyto_ elevation in young MHS subjects alters the activity of calcium‐dependent phosphatases, kinases, and proteases, further impairing glucose and glycogen metabolism in skeletal muscle [9, 10].

In this study, we aim to define the effects of altered resting calcium levels in skeletal muscle, combined with age and sex, on an individual's systemic fasting blood sugar (FBS) levels.

## 2 Methods

### 2.1 Patients and Ethical Approval

Upon institutional research ethics board approval from both University Health network, Toronto, Canada and Rush University, Chicago, US, patients at the Malignant Hyperthermia Investigation Unit (MHIU) were recruited and studied. Patients were diagnosed using the caffeine‐halothane contracture test (CHCT) and studied over the past 10 years. Recruitment criteria included one or more of the following: a prior adverse reaction to anaesthesia, a family history of MH without a confirmed diagnostic mutation (https://www.emhg.org), carrier of a variant of unknown significance (VUS) in *RYR1* or *CACNA1S*, recurrent exercise‐ or heat‐induced rhabdomyolysis, or idiopathic elevation of serum creatine kinase.

The studies presented here include demographic and clinical data from 55 control (MH‐negative) and 86 MH‐susceptible (MHS) individuals, along with [Ca^2+^]_cyt_ measurements in cells derived from muscle biopsies of both control and MH‐susceptible individuals.

### 2.2 Primary Myotube Culture

Human biopsy explants derived from Gracilis muscle were maintained at 37°C in 5% CO_2_/95% air with a growth medium consisting of DMEM/F12 (Millipore Sigma, Burlington, MA, USA), fetal bovine serum 10% (FBS; Foundation; Gemini Bioproducts, West Sacramento, CA, USA), and antibiotic/antimycotic 1% (Gemini Bioproducts). After 10–15 days, cells derived from the explants were transferred by trypsinization to culture dishes for proliferation in growth medium, and myoblasts were expanded through up to four passages. For calcium imaging of myotubes, myoblasts were seeded onto collagen‐coated glass bottom dishes (MatTek, Ashland, MA, USA), and upon reaching 70% confluence, the cells were switched to a differentiation medium (DMEM‐F12 with 2.5% horse serum). Calcium concentrations were measured in live myotubes upon differentiation for 5–10 days.

### 2.3 Cytosolic Calcium Measurements

Resting cytosolic Ca^2+^ concentration, [Ca^2+^]_cyto_, was monitored in myotubes by Shifted Excitation and Emission Ratioing (SEER) of indo‐1 fluorescence [4, 7]. Briefly, for dye loading, cell cultures were immersed in Krebs solution with 5 μM of indo‐1 AM (Invitrogen, Waltham, MA, USA) for 45 min. The Krebs was composed of (in mM) 145 NaCl, 5 KCl, 2.6 HEPES, and 5.6 glucose, pH 7.3. Imaging was by confocal microscopy (scanner TCS SP2; Leica Microsystems; Buffalo Grove, IL, USA), using a 63X, 1.2 numerical aperture water‐immersion objective. SEER of Indo‐1 required the simultaneous acquisition of two confocal fluorescence images produced by alternating line by line two excitation lights (351 and 364 nm) and two fluorescence emission ranges (390–440 nm and 465–535 nm). [Ca^2+^]_cyto_ was derived from fluorescence signals following calibration procedures described elsewhere [11]. Experiments were performed at 20°C–22°C.

### 2.4 Statistical Analysis

All statistical analyses were performed using Sigma Plot and Origin software for Windows. Data are presented as mean ± standard error of the mean (SEM). Differences between group means were assessed using Student's *t*‐test, with a significance threshold set at *p* < 0.05. When data did not meet normality (Shapiro–Wilk test) or equal variance assumptions, the Mann–Whitney Rank Sum test was applied. Distribution violin plots display the mean (blue circle), median (solid line), and the interquartile range (box bounds at the 25th and 75th percentiles). Correlations between [Ca^2+^]_cyto_ and either FBS or age were analysed using Pearson's correlation coefficient and analyses therein. Additionally, multivariate regression analysis was used to evaluate the relationships between [Ca^2+^]_cyto_, FBS, and age.

## 3 Results

Between 2013 and 2024, Rush University received muscle biopsies from 350 subjects who were tested for susceptibility to malignant hyperthermia (MH) with the caffeine‐halothane contracture test (CHCT) at the Malignant Hyperthermia Investigation Unit (MHIU). Of these, biopsies from 152 subjects (73 female and 79 male) were used to establish primary cell cultures for cellular‐level studies. Among these 152 biopsies, 62 (41%) were from MH‐negative (control) subjects, and 90 (59%) were from MHS subjects.

**FIGURE 1:**
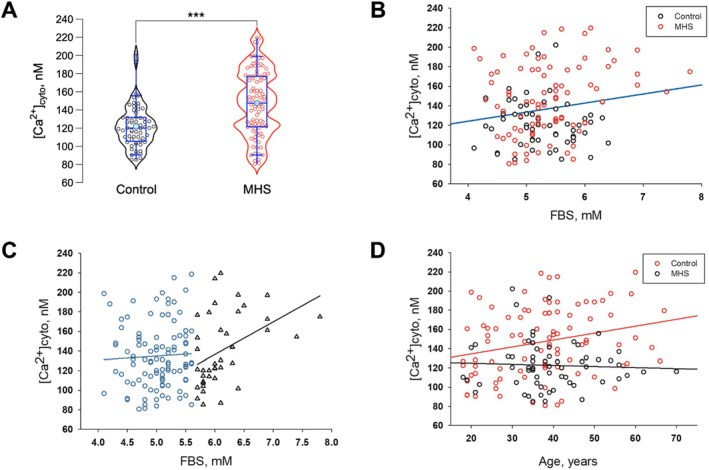
Resting [Ca^2+^]_cyto_ and its association with FBS in Control and MHS subjects. (A) Resting [Ca^2+^]_cyto_ is significantly elevated in myotubes derived from MHS subjects compared to control (MH‐negative) myotubes (*p* < 0.001; 62 MHN, 90 MHS). Each data point represents the average [Ca^2+^]_cyto_ measured from 10 to 30 myotubes. (B) A positive correlation is observed between fasting blood sugar (FBS) levels and resting [Ca^2+^]_cyto_ of primary myotubes (*r* = 0.176; *p* = 0.034; *n* = 145) in entire cohort (blue line) composed of control subjects (open black) and MHS subjects (open red). (C) When the cohort is grouped by FBS levels, individuals with prediabetic FBS (above 5.6 mmol/L, open black triangles) show a strong positive correlation between [Ca^2+^]_cyto_ and FBS (*r* = 0.43; *p* = 0.005; *n* = 40), whereas those with normal FBS (below 5.6 mmol/L, blue open circles) show no significant correlation (*r* = 0.047; *p* = 0.63; *n* = 105). (D) A significant positive correlation is found between the [Ca^2+^]_cyto_ of primary myotubes and the age of the subjects in MHS cohort (*r* = 0.249; *p* = 0.018; *n* = 90), but not in control subjects (*r* = −0.049; *p* = 0.70; *n* = 62). *** indicate *p* < 0.001.

Previous studies have shown that systemic fasting blood sugar (FBS) levels and skeletal muscle cytosolic calcium levels at rest ([Ca^2+^]_cyto_) are markedly elevated in MHS subjects compared to controls [4, 8, 9]. The role of skeletal muscle [Ca^2+^]_cyto_ in glycemic control has been identified in diabetic and MHS mouse models [2]. To further investigate the role of skeletal muscle [Ca^2+^]_cyto_ in human glycemic regulation, we measured [Ca^2+^]_cyto_ in primary myotubes derived from muscle biopsies of MHS and control subjects and analysed correlations among calcium, the individual's FBS levels, age, and sex across the entire cohort. Consistent with our previous observations [4], current data on larger cohorts (illustrated in Figure 1A) feature a significantly higher average resting [Ca^2+^]_cyto_ in myotubes derived from MHS biopsies than in those derived from MH‐negative subjects (147.1 ± 6.18 vs. 122.8 ± 7.5 nM; *p ≤* 0.001). A significant positive correlation, illustrated in Figure 1B, was found between FBS and [Ca^2+^]_cyto_ in the entire cohort (*r* = 0.176; *p* = 0.034) and MHS subjects (*r* = 0.227; *p* = 0.031). Additionally, as shown in Figure 1C, [Ca^2+^]_cyto_ was positively correlated with FBS levels in “prediabetic” individuals (those with FBS > 5.6 mmol/lit; *r* = 0.43; *p* = 0.005), while the correlation was not observed in those with normal FBS (≤ 5.6 mmol/lit; *r* = 0.047; *p* = 0.63).

To further explore age‐associated changes in glucose metabolism, first we studied the association between age and [Ca^2+^]_cyto_. Interestingly, a significant positive correlation between age and [Ca^2+^]_cyto_ is observed in the MHS cohort (*r* = 0.249, *p* = 0.018), as illustrated in Figure 1D, but in contrast no correlation was observed in the control cohort (*r* = −0.049, *p* = 0.70). Our study on a larger cohort of 313 patients, with available FBS values but incomplete calcium data, found a positive correlation between age and FBS in the entire cohort (*r* = 0.129, *p* = 0.022) and as well as in MHS individuals (*r* = 0.185, *p* = 0.030; *N* = 133), supporting the presence of age‐associated changes in glucose metabolism. Regarding sex‐based differences, we found a positive correlation between [Ca^2+^]_cyto_ and FBS in men (*r* = 0.233; *p* = 0.044), but not in women (*r* = 0.085; *p* = 0.47).

**FIGURE 2:**
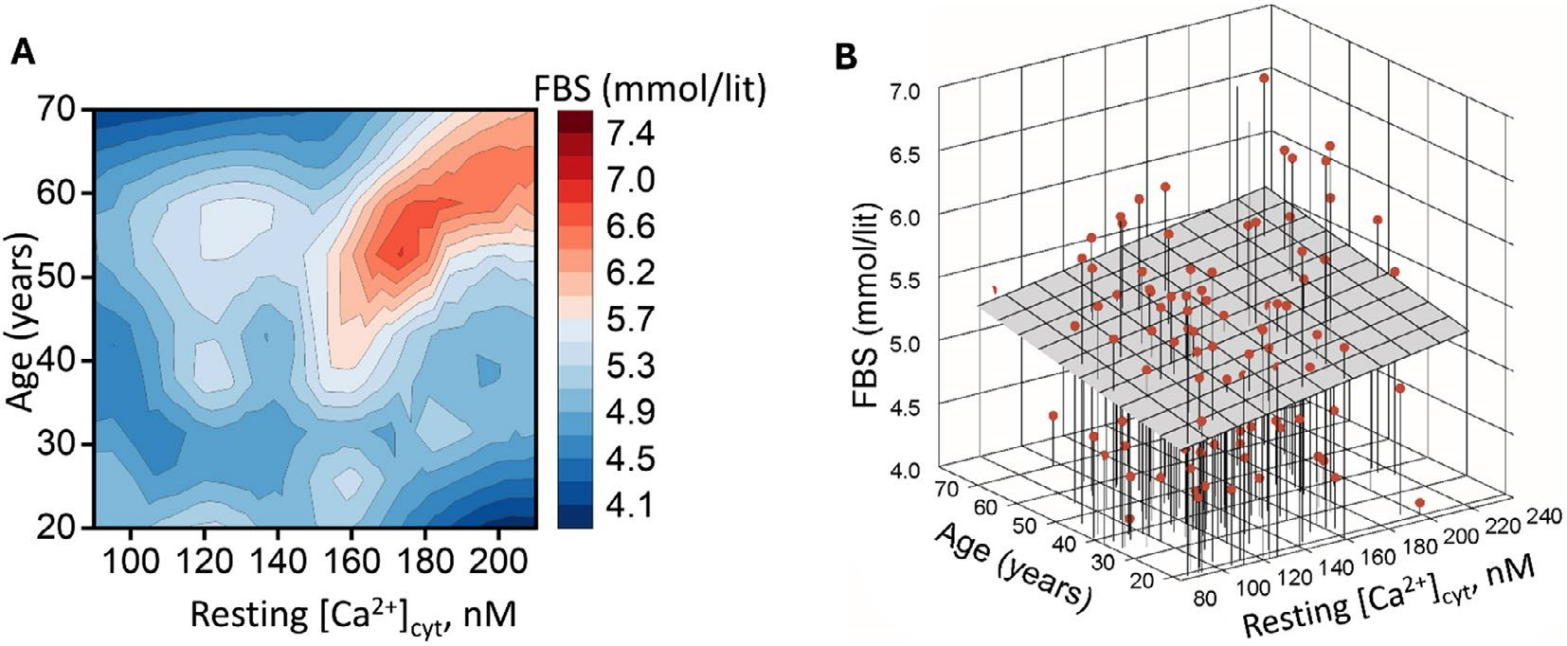
Combined effect of aging and elevated resting [Ca^2+^]_cyto_ on FBS levels. (A) Heat map showing FBS levels in relation to age and resting [Ca^2+^]_cyto_, with prediabetic FBS range (≥ 5.6 mmol/L) shaded in red and normal FBS levels (< 5.6 mmol/L) shaded in blue. Subjects over 40 years with [Ca^2+^]_cyto_ above 150 nM appear to have an increased risk of prediabetes. (B) 3D mesh plot illustrating the relationship between FBS, age, and [Ca^2+^]_cyto_ for each subject with a red headed lane. Best subset regression analysis reveals that the combined effects of increasing age and elevated [Ca^2+^]_cyto_ significantly increase FBS levels (*r* = 0.220), compared with the individual influence of elevated [Ca^2+^]_cyto_ alone (*r* = 0.176; *p* = 0.034) or increasing age alone (*r* = 0.156; *p* = 0.060).

**FIGURE 3:**
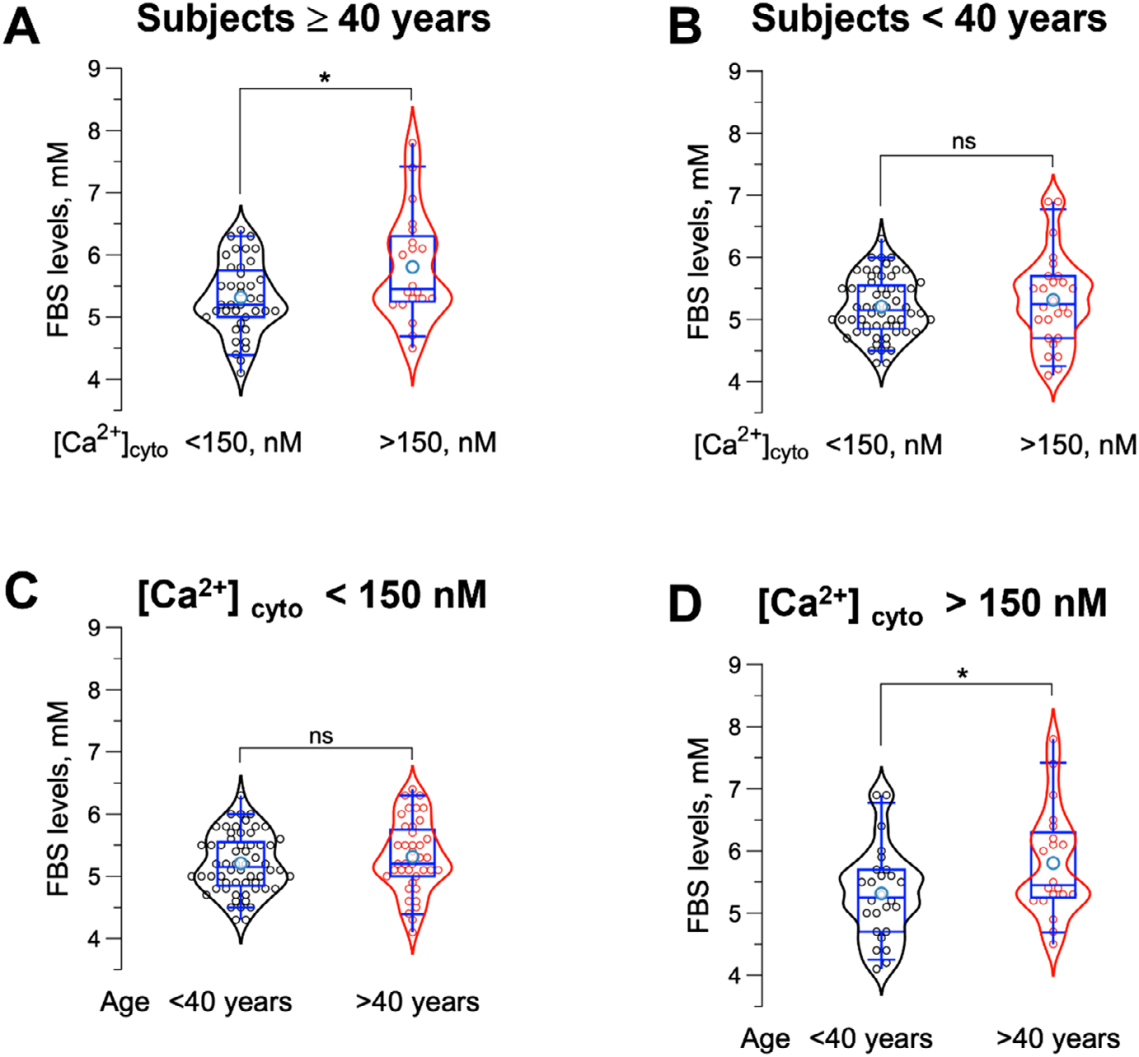
Influence of Age and resting [Ca^2+^]_cyto_ on FBS Levels. (A) Violin‐box plot comparison of FBS levels in older subjects (≥ 40 years) with [Ca^2+^]_cyto_ below and above 150 nM, showing a significant difference (*p* = 0.013; *n* = 20 for > 150 nM and *n* = 39 for < 150 nM). (B) Box plot comparison of FBS levels in younger subjects (< 40 years) with [Ca^2+^]_cyto_ below and above 150 nM, showing no significant difference (*p* = 0.61; *n* = 26 for > 150 nM and *n* = 60 for < 150 nM). (C) Box plots comparing FBS levels between younger (< 40 years) and older (≥ 40 years) subjects with [Ca^2+^]_cyto_ below 150 nM, showing no significant difference (*p* = 0.32; *n* = 60 for < 40 years and *n* = 39 for ≥ 40 years). (D) Box plot comparing FBS levels between younger (< 40 years) and older (≥ 40 years) subjects with [Ca^2+^]_cyto_ above 150 nM, revealing a significant difference (*p* = 0.046; *n* = 20 for < 40 years and *n* = 26 for ≥ 40 years). * indicate *p* < 0.05; ns‐non significant.

To further explore the combined and individual effects of age and [Ca^2+^]_cyto_ on FBS levels, we plotted a heat map (Figure 2A) and 3D mesh plot (Figure 2B) for FBS levels against [Ca^2+^]_cyto_ and age for each subject and performed best subset regression analysis. The heat map (Figure 2A) indicates that subjects over 40 years of age and resting calcium levels above 150 nM are at increased risk of prediabetes (FBS > 5.6 mmol/lit). Best subset regression analysis demonstrated that the combined influence of age and [Ca^2+^]_cyto_ increases the predictability of FBS levels (*r* = 0.219) beyond the sole effect that [Ca^2+^]_cyto_ (*r* = 0.176; *p* = 0.034) or age (*r* = 0.156; *p* = 0.060) as shown in Figure 2B.

In Figure 3, we segregated FBS values by age and [Ca^2+^]_cyto_ levels. Among subjects aged ≥ 40 years, those with [Ca^2+^]_cyto_ above 150 nM had significantly higher average FBS levels compared to those with [Ca^2+^]_cyto_ below 150 nM (5.8 ± 0.20 vs. 5.3 ± 0.09; *p* = 0.013; Figure 3A). In contrast, for subjects younger than 40 years, increasing [Ca^2+^]_cyto_ did not impact FBS levels (5.3 ± 0.14 vs. 5.2 ± 0.06; *p* = 0.61, Figure 3B). Additionally, when [Ca^2+^]_cyto_ remained below 150 nM, age did not significantly influence FBS levels, with older subjects (≥ 40 years) showing similar FBS levels to younger subjects (< 40 years) (5.3 ± 0.09 vs. 5.2 ± 0.06; *p* = 0.32; Figure 3C). However, when [Ca^2+^]_cyto_ exceeded 150 nM, older individuals (≥ 40 years) exhibited significantly higher FBS levels compared to their younger counterparts (5.8 ± 0.20 vs. 5.3 ± 0.14; *p* = 0.046; Figure 3D).

## 4 Discussion

Skeletal muscle, the most extensive insulin‐responsive tissue, plays a key role in systemic glucose homeostasis by facilitating significant glucose uptake from the blood. Molecular disruptions in skeletal muscle can impair insulin signalling, reducing glucose uptake efficiency and leading to hyperglycemia. The diminished insulin responsiveness ultimately can disturb whole‐body glucose homeostasis, contributing to metabolic dysregulation and increased transition to type 2 diabetes [1, 12].

Prior studies demonstrated elevated levels of resting cytosolic calcium ([Ca^2+^]_cyto_) in various tissues of diabetic models [2, 13, 14, 15] and myopathy models with a higher incidence of T2D [16]. Our current study establishes the association between [Ca^2+^]_cyto_ of skeletal muscle cells and fasting blood sugar (FBS) in an age‐dependent manner. First, we extended our previous findings using a larger cohort of myotubes derived from muscle biopsies, and support the observation that [Ca^2+^]_cyto_ in MHS is significantly higher than that in myotubes derived from MH‐negative subjects [4]. Our analysis also revealed distinct patterns of correlation between FBS, [Ca^2+^]_cyto_, and age. Specifically, [Ca^2+^]_cyto_ in primary myotubes showed a positive correlation with FBS in MHS subjects and with the entire cohort, but not within the control group. These observations suggest abnormally elevated [Ca^2+^]_cyto_ in MHS skeletal muscle might contribute to insulin resistance, leading to elevated systemic FBS levels. A significant positive correlation between age and [Ca^2+^]_cyto_ in the MHS cohort may suggest that oxidative stress in the aging process may further exacerbate the RyR1 calcium leak by oxidising these channels [17]. Our further analysis, which demonstrates a differential association between [Ca^2+^]_cyto_ and FBS in males and females, aligns with previous findings suggesting that diabetes manifests differently in men and women [18]. Heat map analysis examining the combined effects of calcium and age identified a clear threshold of [Ca^2+^]_cyto_ at 150 nM and age at 40 years that leads to prediabetic FBS levels. The moderate correlations between fasting blood sugar (FBS) levels and myotubes [Ca2+]_cyto_ levels are expected, as FBS is influenced by multiple systemic and local factors. Our findings suggest that muscle calcium is one of several contributors to systemic FBS regulation.

Further analysis showed that elevated resting calcium did not impact FBS levels in younger individuals (< 40 years), but significantly affected FBS in older individuals (≥ 40 years). Although the increased [Ca^2+^]_cyto_ does not immediately translate to elevated FBS in younger subjects, it may serve as an early indicator for onset of T2D risk in older individuals. In fact, our previous work on skeletal muscle biopsies from young MHS subjects (average age of 35 years) revealed that, despite normal FBS levels, biochemical analyses of these muscle biopsies showed disruptions in glycogen and glucose metabolism enzymes, including increased activities of glycogen phosphorylase, phosphorylase kinase, calcium‐activated calpains, and the calpain‐mediated cleavage and activation of glycogen synthase kinase 3β, alongside reduced GLUT4 and glycogen levels [9, 10, 19]. Elevated insulin levels in young MHS subjects may help regulate FBS within a healthy range, counteracting calcium‐linked irregularities in glucose and glycogen metabolism and help mitigate developing insulin resistance in skeletal muscle [20]. However, in older individuals, high calcium‐induced insulin resistance in skeletal muscle translates to elevated FBS levels because the pancreatic β‐cells' adaptive response to maintain FBS control diminishes with age [21].

It is noteworthy that aging did not correlate with FBS among individuals with [Ca^2+^]_cyto_ below 150 nM. In contrast, the correlation was significant when [Ca^2+^]_cyto_ exceeded 150 nM. These findings highlight the essential role of maintaining calcium homeostasis in skeletal muscle to prevent insulin resistance. Supporting this conclusion, previous studies have shown that treating diabetic mice that have elevated cytosolic calcium using the RyR1 calcium blocker, dantrolene, effectively normalised FBS levels [2].

## 5 Conclusions

Our findings demonstrate a significant role of skeletal muscle resting calcium levels in whole‐body glycemic control. Elevated resting [Ca^2+^]_cyto_ is positively associated with increased FBS, particularly in older and prediabetic subjects. In younger individuals, the correlation between elevated resting [Ca^2+^]_cyto_ and FBS may be obscured by compensatory increases in insulin levels. Notably, when resting calcium in skeletal muscle is maintained below 150 nM, aging does not appear to impact FBS levels, highlighting the potential of [Ca^2+^]_cyto_ modulation at rest as a strategy to manage age‐related glucose dysregulation.

## Author Contributions

**Eshwar R. Tammineni:** conceptualization, data curation, data analysis, investigation, methodology, validation, writing – original draft, writing – review and editing. **Lourdes Figueroa:** conceptualization, data curation, data analysis, investigation, methodology, writing – review and editing. **Michael Fill:** data analysis, validation, writing – review and editing, funding acquisition. **Sheila Riazi:** methodology, data acquisition, data analysis, writing – review and editing, funding acquisition. **Carlo Manno:** data curation, data analysis, investigation, methodology, writing – review and editing.

## Conflicts of Interest

The authors declare no conflicts of interest.
